# On the top of ARB N/L type Ca channel blocker leads to less elevation of aldosterone

**DOI:** 10.1042/BSR20160129

**Published:** 2016-09-16

**Authors:** Tadashi Konoshita, Saori Kaeriyama, Machi Urabe, Takahiro Nakaya, Mika Yamada, Mai Ichikawa, Katsushi Yamamoto, Satsuki Sato, Michiko Imagawa, Miki Fujii, Yasukazu Makino, Yasuo Zenimaru, Shigeyuki Wakahara, Jinya Suzuki, Tamotsu Ishizuka, Hiroyuki Nakamura, for the Genomic Disease Outcome Consortium (G-DOC) Study Investigators

**Affiliations:** * Third Department of Internal Medicine, University of Fukui Faculty of Medical Sciences, Fukui, Japan; † Department of Environmental and Preventive Medicine, Kanazawa University Graduate School of Medical Science, Kanazawa, Japan

**Keywords:** aldosterone, amlodipine, angiotensin receptor blocker, calcium channel blocker, cilnidipine, renin, renin–angiotensin system

## Abstract

The activation of the renin–angiotensin system (RAS) is one of the unfavourable characteristics of calcium channel blocker (CCB). N type calcium channel is thought to be involved in renin gene transcription and adrenal aldosterone release. Accordingly, N/L type CCB has a possibility of less elevation of plasma aldosterone concentrations (PAC) among CCBs. In a monotherapy study, we had already demonstrated that N/L type CCB leads to less activation of the RAS compared with L type CCB. The objective of this study is to substantiate the hypothesis that at the condition of additive administration on the top of an angiotensin receptor blocker (ARB), still N/L type CCB leads to less elevation of PAC compared with L type one. Subjects were 60 hypertensives administered with valsartan. As an open label study, amlodipine (L type) or cilnidipine (N/L type) were administered on the top of valsartan (ARB) in a cross-over manner. Results were as follows (valsartan+amlodipine compared with valsartan+cilnidipine): systolic blood pressure (SBP)/diastolic blood pressure (DBP) (mmHg): 132±10/76±10 compared with 131±10/77±9, *P*=0.95/0.48, plasma renin activity (PRA) (ng/ml·h): 2.41±2.67 compared with 2.00±1.50 *P*=0.20, PAC (pg/ml): 77.3±31.0 compared with 67.4±24.8, *P*<0.05, urinary albumin excretion (UAE) (mg/gCr): 105.9±216.1 compared with 73.9±122.2, *P*<0.05. Thus, PAC at cilnidipine was significantly lower than those at amlodipine in spite of the comparable BP reductions. Besides, UAE was significantly lower at cilnidipine. In conclusion, on the top of the ARB, it is suggested that cilnidipine administration might lead to less elevation of PAC and reduction in UAE compared with amlodipine.

## INTRODUCTION

Calcium channel blocker (CCB) is one of the most useful anti-hypertensive agents in respect of sure anti-hypertensive effect and no crucial adverse effect. However, the activation of the renin–angiotensin system (RAS) is one of the unfavourable characteristics of CCB in a stand point of organ protection, because the RAS plays major roles in blood pressure (BP) regulation and electrolyte metabolism [1], at the same time, the over-activation of the RAS is thought to play pivotal roles in the pathophysiology of cardiovascular [2], renal [3] and metabolic conditions [4]. The activation of the system by CCB is thought to be inevitable mainly via sympathetic nerve activation which activates the human renin gene transcription [5–7]. At the same time, N type calcium channel is thought to be involved in aldosterone release from adrenal cortex [8]. Thus, N/L type CCB, which is classified as the fourth generation CCB according to sympathetic nerve effects [9], has a probability of less activation of the RAS among CCBs. Actually, in a monotherapy study, we had already demonstrated that this type of CCB, cilnidipine, leads to less activation of the RAS compared with L type CCB [10]. And it was shown that this type of CCB, cilnidipine, prevents the reflex up-regulation of plasma aldosterone levels [11].

Recently, for the organ-protective hypertension therapy, angiotensin receptor blocker (ARB) is often selected as the first line agent and CCB is added as a complementary agent. Thus it should be a clinically important issue whether the RAS activation and UAE levels are different among L type CCB and N/L type CCB. Thereby, we substantiated the hypothesis whether at the condition of additive administration on the top of an ARB, still N/L type CCB leads to less activation of the RAS, especially plasma aldosterone concentrations (PAC) compared with L type CCB.

## MATERIALS AND METHODS

### Subjects and treatment

Subjects were recruited from 2007 January to 2013 September. Subjects with age less than 20 years old, secondary hypertension, acute phase disorder and severe organ failure were excluded. We had assessed for eligibility for the study on 68 subjects, who did not obtain the targeted BP (generally 140/90 mmHg) by valsartan alone and enrolled 60 consecutive hypertensives administered with valsartan of our out clinic into the study. All subjects were Japanese. At the out clinic in the morning time, on each occasion, BP was taken at least three readings with an automated digital device (Terumo, ES-H51) at sitting position. If readings varied more than 5 mmHg, additional readings were taken until the last two were close with in the 5 mmHg according to general guidelines for hypertension care. BP measurements were tried to be performed in the same way every visit. The body mass index (BMI) was calculated as the weight in kilograms divided by the square of the height in meters. Metabolic syndrome was diagnosed according to the criteria of the Japan atherosclerosis society. Diabetes mellitus (DM) was diagnosed according to the criteria of the World Health Organization. Dyslipidaemia was diagnosed according to the criteria of the International Diabetes Federation. Chronic kidney disease (CKD) was diagnosed according to the criteria of the Japanese society of nephrology. Diabetic subjects, 53.3% of the total subjects, continued to receive their usual care for diabetes. A target glycated haemoglobin level of less than 7.0% was recommended for all subjects. Arterial hypertension was defined as a systolic blood pressure (SBP) of 140 mmHg or more or a diastolic blood pressure (DBP) of 90 mmHg or more on two separate occasions. The study was undertaken in accordance with the Declaration of Helsinki Principles and informed consent for participation was obtained from individuals. The clinical characteristics at valsartan administration were summarized in Tables 1 and 2.

**Table 1:** Characteristics of subjects at valsartan administration* *Plus–minus values are means ± S.D. †The body-mass index is the weight in kilograms divided by square of the height in meters. ‡Values shown are medians (interquartile ranges).

Characteristics	
Number	60
Age, year	65.6±10.7
Male sex, no. (%)	33 (55.0%)
Body-mass index†	24.8±3.5
Waist circumference, cm	87.2±8.7
Metabolic syndrome, no. (%)	29 (48.3%)
Diabetes mellitus, no. (%)	32 (53.3%)
Dyslipidaemia, no. (%)	34 (56.7%)
Chronic kidney disease, no. (%)	44 (73.3%)
Glucose, mg/dl	134.5±55.3
Glycosylated haemoglobin, %	6.76±1.48
Triacylglycerol, mg/dl‡	107.0 (85.0–144.5)
Cholesterol, mg/dl	
High-density lipoprotein‡	53.0 (42.0–62.5)
Low-density lipoprotein	124.5±30.2
eGFR, ml/min/1.73 m^2^	72.0±19.3
Angiotensin converting enzyme, IU/l	14.9±4.1

**Table 2:** Baseline characteristics and effects of amlodipine and cilnidipine* * Plus–minus values are means±S.D. † The differences between amlodipine and cilnidipine administration on the top of valsartan were analysed by Student's *t* test or Wilcoxon signed rank test as appropriate. Actually differences about urinary data were tested by Wilcoxon signed rank test. All *P* values are two-sided. ‡ *P*<0.05 for the comparison with baseline. § *P*<0.05 for the comparison with the state of valsartan alone. || Values shown are medians (interquartile ranges).

Characteristics	Baseline	Valsartan	Valsartan and amlodipine	Valsartan and cilnidipine	*P* value†
Blood pressure, mmHg					
Systolic	162±18	145±16‡	132±10‡§	131±10‡§	0.95
Diastolic	93±12	87±10‡	76±10‡§	77±9‡§	0.48
Pulse rate, beats/min	73±14	73±12	74±12	73±13	0.32
Serum sodium, mEq/l	141±2	141±2	141±2	141±2	0.49
Serum potassium, mEq/l	4.1±0.3	4.1±0.4	4.2±0.5	4.2±0.5	0.86
Serum chloride, mEq/l	105±3	105±2	106±2	105±3	0.22
Serum creatinine, mg/dl	0.80±0.33	0.79±0.31	0.83±0.35	0.83±0.34	0.94
Urinary sodium excretion, mEq/creatinine||	137 (99–289)	171 (112–271)	150 (94–235)	199 (104–264)	0.05
Urinary potassium excretion, mEq/creatinine||	46 (31–75)	51 (38–76)	45 (36–71)	51 (37–70)	0.80
Urinary chloride excretion, mEq/creatinine||	166 (104–296)	179 (130–294)	168 (102–263)	198 (116–273)	0.16
PRA, ng/ml·h	0.64±0.56	1.50±2.14‡	2.41±2.67‡§	2.00±1.50 ‡§	0.20
Plasma ANP, pg/ml	32.2±17.5	33.3±23.2	38.1±27.5	38.7±25.2	0.79

**Figure 1:**
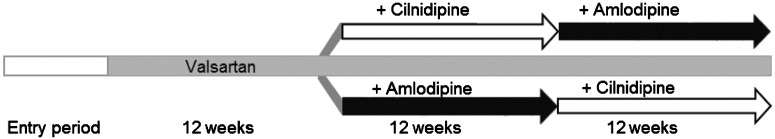
The flow chart of the study of the comparison study of two types of Ca channel blockers on the top of ARB

As an open label study, on the top of valsartan (ARB) administration, the additive amlodipine (L type CCB) or cilnidipine (N/L type CCB) was administered for 12 weeks each in a cross-over manner by randomized turn with a table as a combination therapy without washout periods (Figure 1). Amlodipine was administered from starting from 5 mg and titrated up to 10 mg or cilnidipine was administered from starting from 10 mg and titrated up to 20 mg to achieve a target BP in an intention to treat manner. Actually the subjects were seen twice or thrice in each period and the dose were titrated. No diuretic was administered in the study period. Just before the CCB addition, the items shown in Table 1 were examined. At the complete baseline, sole valsartan administration state and after the additive CCB administration, the items shown in Table 2 were examined. Estimated glomerular filtration rate (eGFR) was calculated according to the formula for Japanese subjects: eGFR (ml/min/1.73 m^2^)=194 × Cr^−1.094^ × Age^−0.287^ (× 0.739, in the case of female). After 15 min rest in the supine position, blood samples were drawn for the measurement at our out clinic in the morning. Ideally sampling should be done on the same time of the day for RAS components measurements. However, there was a limit for actual practice on out clinic. For avoiding cold activation and degradation of humoral factors, the plasma samples were frozen as soon as possible. For plasma renin activity (PRA) measurement sample were incubated 37°C for adequate hours and generated angiotensin I was measured by radioimmunoassay (Bio Medical Laboratories). PAC and atrial natriuretic peptide (ANP) were assayed by radioimmunoassay (Bio Medical Laboratories). Urinary albumin excretion (UAE) was measured by immunoturbidimetry (Siemens Healthcare K. K.).

### Statistical analysis

The sample size of the study was calculated estimating a standard deviation for the PAC of about 25; a difference to be detected between groups of 10 pg/ml and used a bilateral paired Student's *t* test with protection against type I error of 5% and 80% of power. From Altman's nomogram with 2*δ*/*σ*_d_ value for paired *t* test, it was calculated tentatively the study required around 50 subjects in total. Statistical analyses were performed with SPSS Version 22.0 (SPSS Japan). Data were presented as numbers, percentages, means±S.D. or medians (interquartile ranges), as appropriate. The differences between two paired continuous variables were analysed by Student's *t* test fundamentally or Wilcoxon signed rank test appropriately. The difference in PAC and UAE were analysed by repeated measures ANOVA.

## RESULTS

Final doses of amlodipine besilate and cilnidipine were 6.0±2.6 mg/day and 13.0±4.8 mg/day, respectively. On the top of valsartan, a total number of 60 subjects received combination therapy with amlodipine or cilnidipine by turns in a cross-over manner for 12 weeks each, so that the study lasted 24 weeks totally. No serious adverse effect occurred in the study term. Changes in clinical and biochemical characteristics with drugs administration are summarized in Table 2.

**Figure 2:**
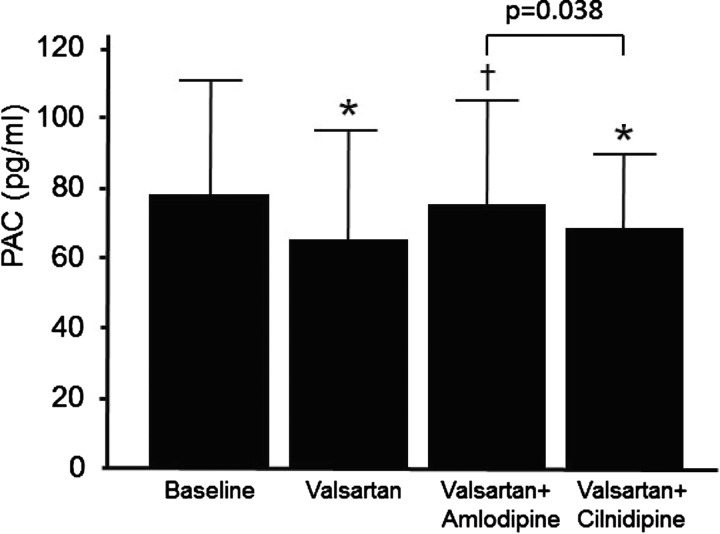
PAC at the endpoint of each CCB administration Closed columns and bars express means and standard deviations of PAC. Significant differences were revealed among treatment status analysed by repeated measures ANOVA (*F*=5.639, *P*=0.001). *P<0.05 for the comparison with baseline. †*P*<0.05 for the comparison with the state of valsartan alone. The exact *P* values from baseline were 0.001 and 0.009 for valsartan and valsartan+cilnidipine, respectively. The exact *P* values from valsartan were 0.006 for valsartan+amlodipine.

**Figure 3:**
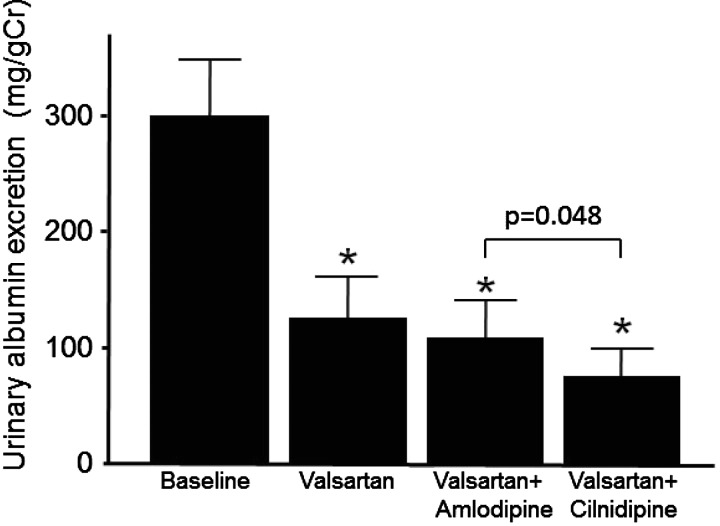
UAE at the endpoint of each CCB administration Closed columns and bars express means and standard errors of UAE. Significant differences were revealed among treatment status analysed by repeated measures ANOVA (*F*=6.981, *P*=0.008). *P<0.05 for the comparison with Baseline. †*P*<0.05 for the comparison with the state of valsartan alone. The exact *P* values from baseline were 0.006, 0.014 and 0.007 for valsartan, valsartan+amlodipine and valsartan+cilnidipine respectively.

At the original baseline, which means the state just before the beginning of anti-hypertensive therapy with valsartan as a representative ARB, BP revealed to be 162±18/93±12 mmHg, retrospectively. With valsartan administration, BP had been significantly reduced to 145±16/87±10. PRA had been significantly augmented from 0.64±0.56 to 1.50±2.14. PAC had been significantly reduced from 78.9±34.6 to 63.1±31.9 (Figure 2). UAE had been significantly reduced from 300.1±85.5 (S.E.) to 114.9±24.6 (S.E.) (Figure 3). In these 60 cases, the aimed BP could not be obtained with valsartan administration only. Accordingly, CCBs were added.

With both of CCBs administration, significant reductions in systolic and diastolic BP were achieved from the state of valsartan monotherapy (Table 2). The BP reductions are comparable between amlodipine and cilnidipine. With regard to humoral factors, significant elevations of PRA from the state of valsartan monotherapy were observed by both CCBs. The PRA at cilnidipine tended to be lower compared with that of amlodipine; however, the difference did not reach statistical significance on this setting (Table 2). On the other hand, the PAC at cilnidipine (67.4±24.8) was significantly lower than that at amlodipine (77.3±31.0) (Figure 1). The PAC at cilnidipine was also significantly lower than the original baseline. Compared with the state of valsartan monotherapy, the PAC at amlodipine was significantly elevated. On the top of valsartan, the UAE at cilnidipine (73.8±16.0 (S.E.)) was significantly lower than that at amlodipine (105.9±28.4 (S.E.)) (Figure 2). Thus, in spite of the comparable BP reductions, significant differences are observed in PAC levels and UAE between the two CCBs.

## DISCUSSION

Recent studies of the human renin revealed the transcriptional mechanism [5–7], the gene expression [12] and genetic and environmental factors [13]. CCB is thought to up-regulate renin gene transcription via catecholamines–β1 adrenoceptor–cAMP–PKA–CREB–CRE pathway [14] and Ca–Ref1–nCaRE pathway [15]. On the other hand, calcium channel is thought to be involved in aldosterone release from adrenal cortex [8]. Thus, CCB is thought to give rise to activation of the whole RAS. Recently Ca channels are categorized into several types, namely, L, N, P/Q, R and T types [16,17]. L type channel is mainly expressed in vascular smooth muscle and regulates vascular tones. On the other hand, N type calcium channel is expressed in the sympathetic nerve ends and regulates catecholamines’ release [18]. Cilnidipine was shown to suppress N type calcium channel in isolated sympathetic nerve [19] and to reduce catecholamine secretion rate [20]. Consequently, it is possible that cilnidipine leads to less activation of the RAS compared with L type CCB. Actually, the less activation was reported in animal models [11,21,22]. In a human clinical study, we had shown that cilnidipine leads to less activation of the whole RAS components compared with amlodipine as a monotherapy [10].

In recent guidelines, ARB is recommended for complicated hypertensives such as DM and CKD. However, in many cases, single ARB therapy is not sufficient. Thus the additional agents are required such as CCB. On the other hand, the variation in PAC levels is shown to be associated with increased mortality independent of major established cardiovascular disease (CVD) risk factors [23] and aldosterone synthase gene is shown to be involved in DM occurrence [24]. Thereby, we substantiated the hypothesis that N/L type CCB still leads to less activation of the RAS compared with L type CCB on the top of ARB. As the primary purpose of this study, the results indicate that the PAC at cilnidipine was significantly lower than that at amlodipine. To our knowledge, until now there is only one report about the PAC levels compared among L type CCB and L/N type CCB by Abe et al. [25]. They compared the PAC levels among amlodipine and cilnidipine administered groups (35 cases each) on the top of ARB (not restricted to single agent) and found that although PRA did not differ, the plasma aldosterone level was significantly decreased in the cilnidipine group. The PAC values were almost comparable to our data. As an explanation, it seems to be plausible that cilnidipine less stimulate the release of aldosterone from mainly adrenal cortex compared with amlodipine in this human setting as shown in animal model [11]. For more precise evaluation other reliable parameters reflecting RAS activity should be ideal. So that we managed to measure plasma angiotensin I and angiotensin II in 16 subjects with paired manner by radioimmunoassay (SRL). The results were as follows (pg/ml, median and 50% quartile); angiotensin I: 145 (98.5, 215.0) at amlodipine and 130 (81.0, 265.0) at cilnidipine (*P*=0.485, Wilcoxon's signed rank test), angiotensin II: 11.5 (10.0, 14.0) at amlodipine and 10.5 (8.5, 12.0) at cilnidipine (*P*=0.244, Wilcoxon's signed rank test). Thus, the results did not reached a statistical significance but a tendency was shown that components of RAS were less activated at cilnidipine indicating that low tendency of PRA might effect on the lower stream of the system cascade. Accordingly, it seems that not only the release of aldosterone from adrenal cortex but also lower PRA partly might contribute to the results.

It has been revealed that UAE is a risk factor for end stage renal disease and CVDs [26,27]. Besides, reductions in UAE are associated with a trend in reducing renal death cardiovascular events [28,29]. As a design of monotherapy with CCB, compared with amlodipine, UAE reduction was observed by cilnidipine in several studies including our previous reports [10,30,31]. Add-on effect of cilnidipine on the top of RAS inhibition on UAE reduction was reported [25,32–37] with conflicting several reports [38,39]. Finally, our study also could indicate that significant UAE reduction was obtained by cilnidipine compared with amlodipine in relatively large scale subjects as an add-on therapy.

Several limitations of this study should be noted. Mixture of non-diabetic and diabetic subjects might have effects on the results. We did not evaluate ambulatory BP results compared with office BP. Considering the earnest practical limit for clinical human subjects in Japan, washout period between the two drugs administration was not set up. The sample number may be still relatively small. Although primary endpoint of the study was changes in PAC from the state of single valsartan administration, we used multiple statistical comparisons. The change in eGFR could be more precise surrogate as well as albuminuria; however, the observation period was not sufficient for its evaluation.

In conclusion, it is suggested that cilnidipine might lead to less elevation of plasma aldosterone level compared with amlodipine for the first time in human clinical study as the primary end point with anti-albuminuric effect. Further investigations should be necessary for evaluation for hard end points.

## Abbreviations

ANP: atrial natriuretic peptide
ARB: angiotensin receptor blocker
BP: blood pressure
CCB: calcium channel blocker
CKD: chronic kidney disease
CVD: cardiovascular disease
DM: diabetes mellitus
eGFR: estimated glomerular filtration rate
PAC: plasma aldosterone concentrations
PRA: plasma renin activity
RAS: renin–angiotensin system
UAE: urinary albumin excretion
